# Phenylhydrazine hydrochloride induced dosedependent embryo cytotoxicity in zebrafish

**DOI:** 10.6026/97320630015255

**Published:** 2019-04-15

**Authors:** Raskin Erusan Rajagopal, Meenakshi Balasubramanian, Shantaraman Kalyanaraman

**Affiliations:** 1 1Multi-Disciplinary Research Unit, Tirunelveli Medical College, Tirunelveli, TamilNadu 627011, India; 2 2Department of Pharmacology,Tirunelveli Medical College, Tirunelveli, TamilNadu 627011, India; 3 3Department of Pathology, Tirunelveli Medical College, Tirunelveli,TamilNadu 627011, India

**Keywords:** Phenylhydrazine hydrochloride, embryo, cytotoxicity, zebrafish

## Abstract

Phenylhydrazine hydrochloride (PHZ) is a chemical compound. PHZ and its derivatives were used firstly as antipyretics, treatment of blood disorders such as polycythaemia Vera. For many years phenyl hydrazine was used for experimental induction of anaemia in animal models. However, this compound is reported to cause damage to red blood cells, potentially resulting in anaemia and consequential secondary involvement of other tissues, such as the spleen and liver. Recent studies suggest that PHZ cause genotoxicity in mice models. The aim of our study is to study the effect of PHZ in embryonic and larval stage of zebra fish model. Zebra fish embryos and larvae were used in this study. Working concentration prepared from 0.05 gm of PHZ stock solution. The embryos and larvae were exposed to different concentrations of PHZ (0.1, 0.3, 0.5, 0.7 0.9, 1.0, 3.0, 5.0, 7.0 9.0 and 10.0 µg/mL) and (0.1, 0.3, 0.5, 0.7, 0.9 and 1.0 µg/mL) respectively. Survival rate, mortality rate, hatching rate and phenotypic anomalies were studied in developing embryos. Heart rate and apoptosis were evaluated to assess the PHZ toxicity in larval stage of Zebra fish. Statistical analysis was performed by Pearson correlation and P values < 0.05 were considered statistically significant. The LC50of PHZ in embryo and larvae was found to be 0.7µg/mL. PHZ treated embryos showed that survival rate was decreased during 72hpf. In the case of mortality, 0.7 µg/mL and above concentration mortality rate was significantly increased between 48 and 72 hpf and the none embryos survived after 72 hpf. We observed delayed hatching rate in treated embryos when compared to control embryos. 0.5 µg/mL treated larvae showed significantly (p<0.05) decreased heart rate 20% at 96 hrs. Phenotype anomalies such as enlarged yolk sac, yolk sac split, pericardial edema, notochord anomaly appeared at higher concentration of PHZ treated embryos. Acridine orange fluorescence staining revealed that high apoptotic cells were detected at caudal fin region of larvae on day 3 at a concentration of 0.7µg/mL treated group. Our study suggests that PHZ causes multiple phenotypic abnormalities and toxicity on zebrafish embryos and larvae with respect of dose and time dependent manner.

## Background

Phenylhydrazine hydrochloride (C6H8N2.Hcl) is a compound, primarily used as a intermediate in the pharmaceutical, agrochemical, and chemical industries 1. Phenylhydrazine hydrochloride (PHZ) and its derivatives were used as antipyretic drug 2, while it has been reported to cause decrease in hemoglobin levels, red blood cell counts and packed cell volume and increases in mean cell volume, mean corpuscular hemoglobin and mean corpuscular hemoglobin concentration. It is also reported to cause extramedullary haematopoiesis in the spleen and liver 3. PHZ is also known to induce oxidative stress on erythrocytes resulting in oxidation of oxyhemoglobin, formation of methemoglobin, conversion into irreversible hemichromes, precipitation of hemoglobin in the form of Heinz bodies and hemolytic anemia 4. PHZ also causes cytoskeletal proteins denaturation, lipid peroxidation, ATP depletion, cation imbalances, and reduced membrane deformability 5. This study is aimed to analyse the effect of phenyl hydrazine hydrochloride on developing embryos and larval stage of zebrafish (Danio rerio).

Zebrafish is an experimental model organism offering many advantages. Its embryos are externally fertilized and possess optical transparency which makes it easy to detect in real-time morphological endpoints and observe developmental processes in its early life stages under light microscopy. Zebrafish is also one of the most promising in vivo models for assessing drug effects and toxicity studies 6 due to its genetic, physiologic, and pharmacologic similarity with human beings 7 and also because of its small size, low cost of maintenance and easy breeding 8. This high fecundity has made large-scale chemical and genetic screening possible with the embryos or larvae exhibiting a diverse range of biological processes and fully integrated vertebrate organ systems 9. In this study, zebrafish embryo and larva was used to document the toxic effects of phenyl hydrazine, through estimation of the phenotypic anomalies, apoptosis, heart rate, hatching rate, survival and mortality rate.

## Methodology

### Chemicals:

Phenylhydrazine hydrochloride was procured from Sisco Research Laboratories Pvt. Ltd, while Dimethyl sulfoxide (DMSO), MS-222 (tricaine methanesulfonate) and acridine orange were procured from Sigma Aldrich Inc. All chemicals and reagents were of the molecular biology grade.

### Experimental animals:

Healthy wild-type adult zebrafish were procured from a local commercial breeder for the study. All the procedures were approved by the Institutional animal ethics Committees. The fish were maintained at the laboratory in 50 litre tanks separately for male and female at 27±2°C with constant light and dark (14-10 hrs) cycles. They were fed twice a day with a commercial food pellets (Inch-Gold tropical food and dried blood worms). The health condition of the fish was checked regularly every day 10.

### Breeding:

Prior to spawning, males and females were housed separately for a minimum of 3 days. The day before eggs were required, males and females were placed in breeding tanks with a 2:1 (male-female) ratio. The breeding tanks were equipped with a spawning tray, which consists of a fine net with an appropriate mesh size for eggs to fall through, close to the bottom of the tank. The fish were left undisturbed overnight and eggs could be collected 1 h after the light had been turned on the next morning 11.

### Embryo Collection:

Embryos were collected from breeding tray by using a pasteur pipette and were rinsed with embryo medium. Zebrafish embryos were staged hours post fertilization (hpf) by direct observation under light microscope, based on morphological characteristics described by Kimmel et al (Year) 12. The embryo plates were incubated in embryo medium (EM) at 28±1°C and the pH of embryo medium was checked for all solutions and adjusted to 7.2.

### Phenyl hydrazine hydrochloride treatment:

PHZ stock solution prepared by 0.05gm PHZ dissolved in 10 ml of EM and by diluting various concentration of working solution prepared from the stock solution. Twenty embryos of 6 hpf were transferred to 6 well flat bottom plate and each well contained 3 ml of EM. Embryos were exposed to different concentrations of PHZ (0.1, 0.3, 0.5, 0.7 0.9, 1.0, 3.0, 5.0, 7.0 9.0 and 10.0 µg/mL) for 72 hpf. Simultaneously controls embryos treated with EM alone. Both treated and control embryos were incubated at 28±1°C. We examined the embryo development periodically, every 24 hrs and recorded survival rate, hatching rate and phenotypic abnormalities using an Inverted microscope (Radical -RTC-7).

To determine the larval toxicity, twenty free-swimming larvae (4day) were used. The larvae were transferred to 6 well flat bottom plates with each well containing 3 ml of embryo medium. Larvae were exposed to various concentration of PHZ (0.1, 0.3, 0.5, 0.7, 0.9 and 1.0 µg/ml) and incubated at 28±1°C. We documented the larval development at 24 hour intervals including the survival rate, heart rate and mortality. The larvae that failed to respond to strong tactile stimuli were considered dead and were removed immediately. The mortality of larvae was recorded at 24, 48, 72, and 96 hrs postexposure at each concentration of the PHZ. The median lethal concentration (LC50) was determined with the mortality data.

### Survival and hatching rates:

The embryos were kept on 4 hourly observations under an inverted microscope for documenting mortality, hatching, delayed growth and phenotypic abnormalities like skeletal malformations, pericardial edema, and yolk sac split. The percentage of survival and hatching rate was defined as the number of larvae survived /the initial number of embryos) x 100 13.

### Heart rate (HR) measurement:

To determine the effects of PHZ on heart rate, 72 hpf larvae were incubated in various concentrations (0.1, 0.3, 0.5, 0.7, 0.9 and 1.0 µg/ ml) of PHZ and the heart beats per minute was recorded at 24, 48, 72 and 96 hours of treatment. Each heart rate measurement was done by counting the contractions of either of the two chambers for a minimum of 60 secs. Heart rate counting was repeated three times for each embryo and the average was calculated 14.

### Acridine orange staining:

To clarify whether the reduction of cell viability and toxicity at high concentrations of PHZ is related to apoptosis, we stained the 72 hpf embryos with acridine orange. The larvae were exposed to various concentrations of PHZ (0.3, 0.5 and 1.0 µg/ml) up to 3 days. From each set of larvae, one placed in different concentrations of PHZ and the other being untreated controls, 10 larvae were selected and incubated with 5 µg/ml of acridine orange for 30 minutes at room temperature in the dark. Then they were washed thrice with EM solution for 5 minutes each and the live larvae anesthetized with 0.02% tricaine mesylate and mounted laterally on a microscope slide. The slides were then subjected to fluorescence microscopic examination (Radical RTC-7) and all embryos were examined and photographed and the staining patterns were compared. 15-16

### Statistical analysis:

All experiments were repeated 3 times for greater statistical significance, and linearity of the data was tested with Pearson correlation and p values < 0.05 were considered statistically significant. Statistical analysis was performed using SPSS 16.0 software.

## Results

### Effects of PHZ on Survival and Mortality of Zebrafish Embryo:

**Figure 1:**
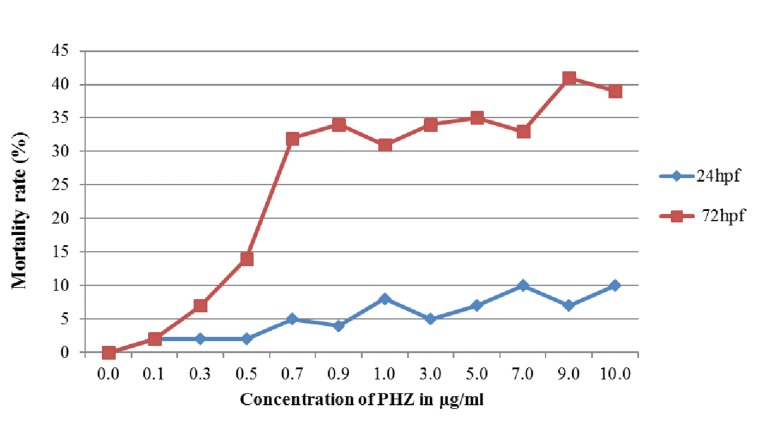
Embryos (at 50 % epiboly stage) were incubated in various concentrations of PHZ and the moratlity rates were recorded at 24 nd 72 hpf. Mortality rate of embryos increased in concentration dependent manner. The death of embryos was defined as no visual heart beat.

**Figure 2:**
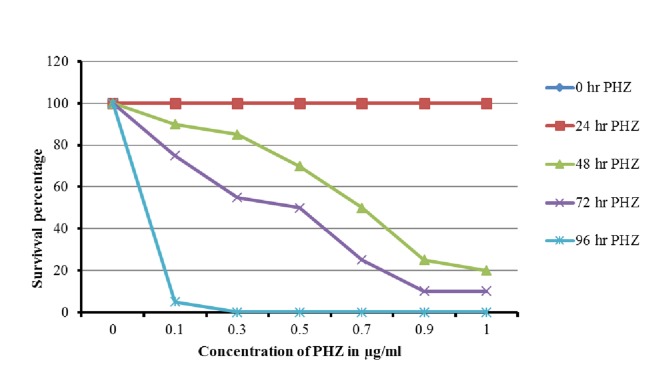
72 hpf larve were incubated in various concentrations of PHZ and the survival rates were recorded at 24, 48, 72 and 96 hr. Survival rate of larvae deveased in concentration dependent manner. The death of an larvae was defined as no visual heart beat and no motality. In Figure 2, survival percentage may changed to Survival rate (%)

After induction with PHZ, at 24 hpf, there was no significant difference in survival rates between PHZ treated and untreated control embryos in both lower (0.1-1.0 µg/mL) and higher (3.0- 10.0 µg/mL) concentrations with a survival of 98% embryos at lower concentration and 90% embryos at higher concentration. The survival rate decreased at 72 hpf with a high mortality in concentrations of 0.7 µg/ml and above, while none of the embryos survived after 72 hpf (Figure 1). The effect of PHZ on larvae (72 hpf larvae) at 0.1, 0.3, 0.5, 0.7, 0.9 and 1.0 µg/ml were tested. PHZ treated larvae and control larvae were incubated at 28 ± 1°C upto 96 hrs. While there were no morphological changes, dead larvae were observed between 0 and day 1 and the mortality increased with increase in concentrations of PHZ between days 3 and 4. We observed that survival rate gradually decreased on day 2 and 3 at low concentrations (0.3-0.5 µg/mL). On day 4, none of the larvae survived at concentrations of 0.7 µg/mL and above. There was no mortality observed in the control larvae throughout the test. This indicates that PHZ has both a time dependent and dose dependent toxicity in zebrafish larvae (Figure 2).

### Hatching Rate:

Zebrafish embryo which normally hatches out at 48 hpf to 72 hpf, when treated with PHZ showed delayed hatching rate compared to the control group. At lower concentrations of PHZ (0.1, 0.3 and 0.5 µg/ml) hatching was seen up to 96 hpf, beyond there was no survival. However, at a concentration of 0.7 µg/ ml and above embryos showed a 50% hatching rate at 72 hpf and none of embryos hatched at 96 hpf (Table 1). This indicates that PHZ decreases the hatching rate of zebrafish embryos when compared to control embryos.

### Heart Rate (HR):

The effect of PHZ on heart rate of test and control zebrafish larvae upto 72 hpf was observed and recorded. Post exposure to PHZ at 24 hpf, the heart beat gradually increased from 60 to 70 beats per minute as the concentration increased from 0.1 to 1.0 µg/ml of PHZ, indicating a dose dependent response, while at 72 hpf, in concentrations of 0.1, 0.3 and 0.5 µg/ml of PHZ, the heart beat slightly decreased compared to 48 hpf. At 96 hpf, heart beats shows significant (p=0.05) decrease at concentration of 0.1, 0.3 and 0.5 µg/ mL of PHZ respectively. In the control larvae heart beats were not decreased throughout the study (Table 2). This indicates, PHZ causes decrease of heart rate which is dose and time dependent.

### Phenotype abnormalities:

**Figure 3:**
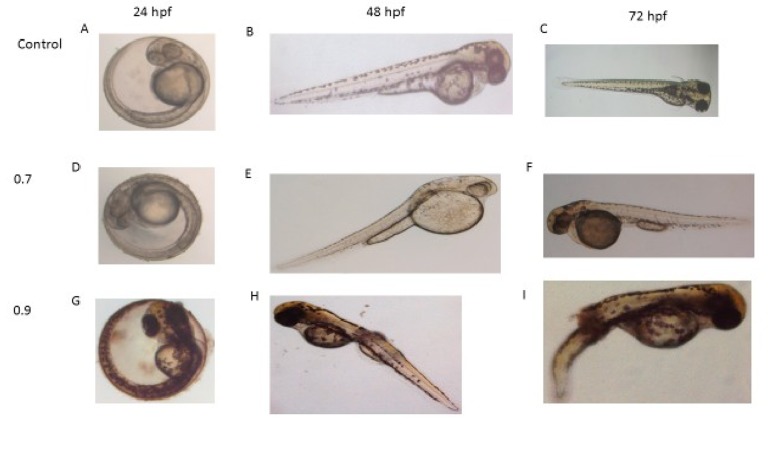
Morphological features of Phenylhydrazine hydro choloride treated embryos at 24, 48 and 72 hpf. (A, B and C) control group, E-enlargement of yolk sac, F-yolk sac split, H notocard anomaly, I- Whole body curvature (arrow). In Figure 3, Arrow mark missing, So, I have attached corrected version of figure 3 (with arrow)

**Figure 4:**
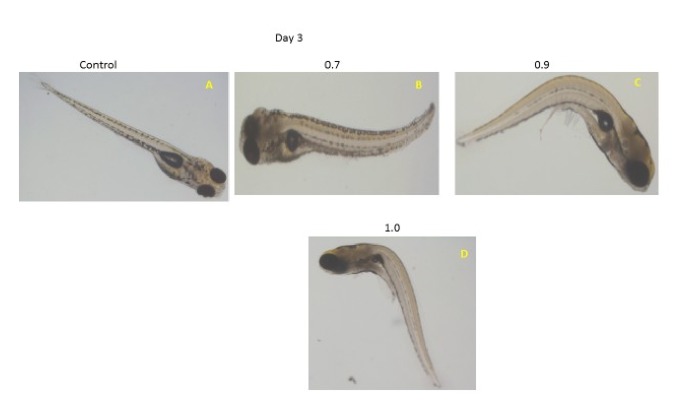
Morphological features of Phenylhydrazine hydro choloride treated larvae on Day 3. A � Control, B � tail bend, C- body curvature and D- severe notocard anamaly (arrow). In Figure 4, Arrow mark missing, So I have attached corrected version of figure 4 (with arrow)

Control embryos were observed to have a normal development as shown in Figures 3 (A-C) whereas PHZ treated embryos showed moderate to severe phenotypic changes during development as shown in Figures 3 (D -I). At 48 hpf, yolk sac enlargement was observed at 0.7 µg/ ml of PHZ Figure 3 (E). Enlargement of yolk sac and yolk sac split were observed at 72 hpf as shown in Figure 3 (F). At 0.9 µg/ ml treated embryos showed multiple malformations such as notochord anomaly, yolk sac split, edema at heart region and under development of eye at 48 hpf. At 72 hpf showed severe phenotype abnormalities like body curvature, tail bend and underdeveloped head as shown in Figure 3 (H-I). PHZ treated larva on the day 3 showed whole body curvature, which gradually increased, and eye size reduced when PHZ concentration increased (Figure 4). This indicates that PHZ causes severe phenotypic anomaly in both treated embryos and larvae. Hence, PHZ is toxic for zebrafish embryos being both dose and time dependent.

### Acridine orange staining:

**Figure 5:**
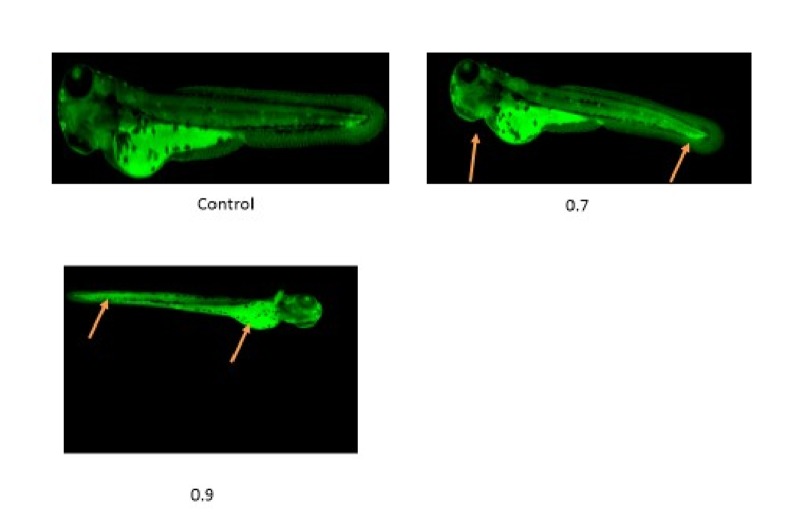
Control embryos showing no apoptotic cells whereas 0.7 �g/ ml treated embryos shows few apoptotic cells in Heart and Tail region, 0.9 µg/ml treated embryos shows increased apoptosis in tail and yolk sac region (arrow).

Results of acridine orange staining showed no apoptotic cells in control larvae but PHZ treated larvae on day 2 showed intense staining at heart, yolk sac and tail region at the concentration of 0.7 µg/ ml. We found high intense staining toward yolk sac to caudal fin region at 0.9 µg/mL treated larvae. High intense acridine orange stain on zebrafish larvae clearly indicates that there was high apoptotic cells present due to the effect of PHZ (Figure 5).

##  Discussion

PHZ and its derivatives are used as antipyretics drug and also to treat blood disorders such as Polycythemia vera 2. However, the mechanism of action of these chemicals is not clear but presumed to have influence on antioxidant system on erythrocytes 17. According to recent study, phenylhydrazine is a hemolytic agent that induces the RBCs lysis leading to drastic reduction in the hematocrit (Hct) and Hb concentration 18. Zebrafish embryo is a good model for detecting environmental contaminants and also toxic effect of chemical substances. We assumed that treating redblooded notothenioid with PHZ would induce severe anemia that could provide insight into toxic effect on developing embryo. The present study evaluates the effect of various concentration of PHZ in both embryo and larvae of zebrafish by observing the phenotypic malformations. The embryos and larvae treated with various concentration of PHZ showed dose and time dependent multiple phenotypic malformations including yolk sac enlargement, under developed eyes and spinal curvature. Apart from that there is a significant (p=0.05) decrement in heart beat (45 beats/minute) and hatching rate (50%) has been observed in zebra embryos treated with 0.5 µg/ mL of PHZ at 96hpf. Similarly, multiple mal formation has been observed in zebrafish larvae exposed to Perfluoroalkyl acids (PFAAs) 19. A possible mechanism for the deformation of the spine or body curvature may be due to apoptosis and alteration in muscle fibers, as attributed previously in perfluorooctane sulfonic acid (PFOS) exposed zebrafish larvae 20. Previous reports evidenced that pericardial edema is also a sign of compromised cardiac output, which was observed in Trifluoroacetic acid (TFAA), perfluorobutyric acid (PFBA), perflurobutane sulfonic acid (PFBS) and perflurooctane sulfonic acid (PFOS) exposed embryos 21. Therefore, the malformations caused by the PHZ could be the result of several mechanisms reinforcing each other. Hatching is a critical phenomenon that depends on digestions of chorion by hatching gland enzymes, and the movement of the embryo to open it. A deleterious effect on any of the processes can delay or inhibit the hatching 22. Reduction in survival rate indicate that the induction of acute toxicity unveiled by supra molar levels of PHZ. The apoptotic potential of PHZ has also been evaluated using acridine orange assay. The results revealed high apoptosis in the yolk sac, heart and predominantly in caudal fin region. This study reports novel data on dose and time dependent effects of PHZ on both embryo and larvae. Together all the observations clearly demonstrate the PHZ could act as toxic compound at microgram level during the embryo development. Further studies are required to find the chemical compound structure and function relationship and mode of action of PHZ.

## Conclusion

This study demonstrates that the zebrafish is a viable model for screening small molecules and its toxicity effects during embryonic development. We also strongly suggest that phenylhydrazine hydrochloride might be toxic for both zebrafish embryo and larva with respect of dose and time dependent manner.

## Conflict of Interest

Authors declare no conflict of interest.

## Figures and Tables

**Table 1:** Relationship of PHZ concentration and Hatching rate of embryos (%)

Concentration of PHZ (µg/ml)	48 hpf	72 hpf	96 hpf
Control	60%	100%	-
0.1	20%	59%	99%
0.3	20%	59%	90%
0.5	20%	58%	80%
0.7	10%	50%	-

**Table 2:** Relationship of PHZ concentration and heart rate of embryos (%)

Concentration of PHZ (µg/ml)	24 hpf	48 hpf	72 hpf	96 hpf
Control	82	98	96	96
0.1	60	59	55	55
0.3	64	58	52	50
0.5	68	55	50	45
0.7	69	56	-	-
0.9	69	58	-	-
1	70	57	-	-
